# Nanoparticles Addition in PU Foams: The Dramatic Effect of Trapped-Air on Nucleation

**DOI:** 10.3390/polym13172952

**Published:** 2021-08-31

**Authors:** Beatriz Merillas, Fernando Villafañe, Miguel Ángel Rodríguez-Pérez

**Affiliations:** 1 Cellular Materials Laboratory (CellMat), Condensed Matter Physics Department, Faculty of Science, Campus Miguel Delibes, University of Valladolid, Paseo de Belén 7, 47011 Valladolid, Spain; marrod@fmc.uva.es; 2 GIR MIOMeT-IU Cinquima-Química Inorgánica, Faculty of Science, Campus Miguel Delibes, University of Valladolid, Paseo de Belén 7, 47011 Valladolid, Spain; fernando.villafane@uva.es; 3 BioEcoUVA Research Institute on Bioeconomy, University of Valladolid, Paseo de Belén 7, 47011 Valladolid, Spain

**Keywords:** air nucleation, cell nucleation density, nanoclays, polyurethane foams, nucleation mechanisms, air trapping

## Abstract

To determine the effect of nanoclays and trapped air on the formation of rigid polyurethane foams, three different production procedures were used. To study the influence of mixing at atmospheric pressure, two approaches were carried out employing either an electric or a magnetic stirrer. The third approach was executed by mixing under vacuum conditions with magnetic stirring. The samples thus obtained were characterized, and the effect of trapped air into the reactive mixtures was evaluated by analyzing the cellular structures. Different levels of trapped air were achieved when employing each manufacturing method. A correlation between the trapped air and the increase in the nucleation density when nanoclays were added was found: the cell nucleation density increased by 1.54 and 1.25 times under atmospheric conditions with electric and magnetic stirring, respectively. Nevertheless, samples fabricated without the presence of air did not show any nucleating effect despite the nanoclay addition (ratio of 1.09). This result suggests that the inclusion of air into the components is key for improving nucleation and that this effect is more pronounced when the polyol viscosity increases due to nanoclay addition. This is the most important feature determining the nucleating effect and, therefore, the corresponding cell size decreases.

## 1. Introduction

Reducing energy consumption in buildings through thermal insulation is one of the major concerns in order to deal with the increasingly demanding environmental normative [1]. A great variety of materials with high insulating capacities are employed to face this problem. Some recent solutions in this area are based on renewable materials, such as wood fiber, blown and sprayed cellulose, wool, hemp, and straw, among others [2,3,4,5,6,7]. However, the conventional insulation materials mostly employed in the construction sector are mineral wools and polymeric foams [8]. Closed-cell polymeric foams are used often due to their low weight, cost-effectiveness, and reduced thermal conductivities [9,10]. Polyisocyanurate (PIR) and polyurethane (PU) foams, based on the reaction between isocyanate and polyol, are two of the most versatile materials employed in a wide range of applications in different sectors, such as building, automotive, and furniture [11]. Particularly, rigid PU (PUR) foams provide effective thermal insulation with typical thermal conductivity values between 0.020 and 0.035 W m^−1^ K^−1^ [12,13,14,15].

Although these PU-based materials are efficient thermal insulators, there is still some margin to improve their performance. Taking into account the heat transfer mechanisms in porous media [16,17], reducing the cell size seems to be an effective way to minimize the radiation contribution to the total thermal conductivity. One commonly used strategy to reduce the cell size is the incorporation of fillers into the PU foams as nucleating agents. These particles can also enhance some properties of the polymeric matrix, such as its stiffness, strength, and flame retardancy [18,19,20,21]. Selecting nanoparticles as fillers for these materials is a logical approach, considering that the cell walls of low-relative-density PU foams (relative density below 0.05) have a low thickness in the range of 0.5 to 1.5 microns [22,23,24]. These nanofillers can be based on a great variety of materials, such as fumed silica [25], sepiolites [26], glass fiber [27], carbon-based nanoparticles [28], or montmorillonites [29] to name a few. Nanoclays are nanoparticles of layered mineral silicates. Furthermore, their surfaces can be functionalized, giving rise to organically modified nanoclays (organoclays) with potential uses in polymer nanocomposites when uniformly dispersed. A wide range of nanoclays have been used in polyurethane foams for different purposes. Thi et al. [21] added the organoclay Cloisite^®^ 20A (2.5 wt%) to expandable graphite (EG)/polyurethane composites, proving that these nanoparticles enhance thermal insulating properties and flame retardancy. Saha et al. [30] employed 1 wt% of platelet nanoclays as fillers in PUR foams, reaching a significant improvement in thermal and mechanical properties, as well as a cell density increase with a consequent reduction in cell size. Kim et al. [31] used Cloisite^®^ 30B, a natural montmorillonite modified with a quaternary ammonium salt, to fabricate PUR/clay nanocomposites. Their results show that an increasing number of particles contribute to decreasing both the cell size and the thermal conductivity of the final foam. Cao et al. [32] also observed this effect on polyurethane/montmorillonite nanocomposites employing 5 wt% of organomodified Closite^®^ 30B, leading to better dispersion and compatibility with the polyol. There are other works that also used these organoclays, such as those of Thirumal et al. [33], Pardo-Alonso et al. [34], and Kang et al. [35]. The later examined the effects of adding Closite^®^ 30B (1, 2, and 3 wt%) and other solid additives to rigid polyurethane foams. Their results suggest that the insulation properties are determined by both the cell size and the number of closed cells. Santiago-Calvo et al. [36] studied the effect of a 5 wt% content of the natural montmorillonite Cloisite^®^ Na^+^ and the organomodified Cloisite^®^ 30B on the microstructure, reaction kinetics, mechanical properties, and thermal conductivity of rigid PU foams. In this case, the thermal conductivity is reduced due to the reduction observed in the cell size, which promotes a decrease in the heat transfer by radiation. The authors report that the nanoclays used in the study might be acting as nucleating agents during the formation of the foams. The presence of the particles alters the homogeneous cell nucleation owing to the physical and chemical interactions with the polymer matrix and the gas phase, as Estravís et al. previously demonstrated by X-ray diffraction measurements and by FTIR studies [37]. 

This interpretation is generally accepted in the literature, and it is based on the fact that the nanoclays introduced in the reacting system also modify the nucleation process, thus inducing a higher number of cells. Imran et al. [38] also demonstrated that the cell size of PU foams decreases for high weight fractions of alumina nanoparticles and, additionally, tensile strength increases. However, the real underlying mechanisms behind this increment in the number of cells has not been discussed in detail. 

In all these reports, the production of the PU foams containing nanoclays was carried out using a high-shear mixing device that, as is well known, introduces air bubbles during the mixing process. In addition, it is also known that heterogeneous nucleation is also caused by the air bubbles introduced during the mixing of the urethane foam components [39,40,41]. Brondi et al. [42] studied the influence of air bubble inclusion on rigid PU and PIR foams. The nucleating effect of air when included during the mixing step at a fast rate was demonstrated. However, this effect is not present when the mixing is performed at slower rates.

Moreover, it is also possible that a polyol with a higher viscosity may trap air in a more effective way during the mixing process. These facts lead us to consider one question not previously analyzed in the literature regarding the cell size reduction in systems containing fillers, that is, the real effect of the presence of particles on the nucleation process. There are two possible reasons for the cell size reduction induced by the presence of particles. The first one is that these particles may reduce the surface tension, thus increasing the number of cells created when CO_2_ is produced from the reaction of isocyanate and water. Nevertheless, effective nucleation could also be due to an alternative mechanism: an increase in the viscosity of the polyol blend containing particles might promote more effective air trapping during high-shear mixing and, as a consequence, more effective nucleation owing to the presence of a higher number of air bubbles in the reacting system. So far, we have not been able to find previous papers studying which mechanism of the two possible options discussed in this paragraph is more effective in reducing the cell size in PU foams containing particles.

This work is focused on addressing this aspect by studying PU foams containing nanoclays as a model system. To discover the real reason behind the nucleation effect of the clays, specific experiments were performed by either promoting the trapping of air in the reactive system or avoiding it by mixing the reactants under vacuum. In addition, viscosity measurements of the different blends were carried out in order to explain how the increase in the viscosity of the blend with nanoparticle inclusion may affect cell nucleation and the final cell sizes. A complete characterization of the cellular structure was performed by evaluating the reductions in the cell size that take place both at atmospheric pressure and under vacuum conditions. 

## 2. Materials and Methods

### 2.1. Materials

The polymeric methane diphenyl diisocyanate (PMDI) was obtained from BASF. Alcupol^®^ R4520, a commercial polyether polyol, was supplied by Repsol, S.A. Distilled water was used as a blowing agent. The main characteristics of this polyol are included in Table 1. *N*,*N*,*N*′,*N*″,*N*″-pentamethyl diethylene triamine (PMDETA) and *N*,*N*-dimethylcyclohexylamine (TEGOAMIN^®^ DMCHA) obtained from Evonik Nutrition & Care GmbH (Germany) were used as catalysts for the blowing and gelling reactions, respectively. Polyether dimethyl siloxane (TEGOSTAB^®^ B 8522) supplied by Evonik Nutrition & Care GmbH (Germany) was used as a surfactant. Organically modified Cloisite^®^ 30B nanoclay (density = 1.98 g cc^−1^, particle size = 2–13 µm, cation exchange concentration = 90 meq/100 g clay, methyl tallow-bis-2-hydroxyethyl quaternary ammonium (MT2EtOH)) and a non-treated montmorillonite Cloisite^®^ Na^+^ (density = 2.86 g cc^−1^, particle size = 2–13 µm, cation exchange concentration = 92.6 meq/100 g clay) obtained from Southern Clay Products (Gonzales, TX, USA) were used as fillers in the formulation. The formulation used to produce the foam is included in Table 2, and the main characteristics of the nanoclays are given in Table 3. The total mass of the foams produced was fixed at 10 g, and the wt% of particles was added over this mass.

### 2.2. Fabrication Method

The organoclays were first dried under vacuum for at least 24 h at 80 °C to remove all traces of moisture. The PU foams samples were fabricated in plastic cups employing different stirring methods, as explained below. Three fabrication methods were implemented. 

This first method corresponds to samples E-A, which refer to the type of stirrer (electric) and pressure conditions (atmospheric). The polyol, water, surfactant, and catalysts were mixed for 5 min using an IKA Eurostar Power control-visc P1 equipped with a Lenart-disc (5 cm diameter) at 500 rpm. Once the blend was homogenized, organoclays were added and mixed for 5 min at 500 rpm with the same stirring equipment. Finally, the isocyanate was added to the mixture, and after 15 s, both components were stirred using the same mixer at 800 rpm for 10 s. 

The second method was designed as M-A, corresponding to the stirrer type (magnetic) and pressure conditions (atmospheric). The first difference with respect to the previous method was the nanoclay-mixing step, which was carried out with a magnetic stirrer for the same time and at the same rate. Then, the isocyanate was added, and after 15 s, the components were mixed with the magnetic stirrer at 800 rpm for 10 s. 

The reference for the third method was M-V, as the mixing to produce these foams was carried out using a magnetic stirrer under vacuum conditions. The first step of mixing the main components was carried out with the electric stirrer under the same conditions as before. When nanoclays were added to the mixture, a magnetic stirrer was used to mix the nanoclays with the polyol blend under vacuum conditions (0.06 bar), thus avoiding additional air trapping in the polyol blend containing the nanoclays. After 5 min of mixing, the isocyanate was added and vacuum (0.06 bar) was again applied before mixing the isocyanate with the polyol blend. Both components were stirred for 10 s at 800 rpm. 

All foams obtained by these three methods were allowed to grow by the free-rise method at atmospheric pressure.

Two different amounts of nanoclays were included in the formulations: 0.5 and 1 wt% of the total mass without particles. The NCO/OH ratio in the formulation was 1.83/1.0 for all the foams produced. After 48 h of curing time at room temperature, the samples obtained were cut for characterization.

The nomenclature and experimental characteristics of all the samples in this study are summarized in Table 4.

### 2.3. Characterization

#### 2.3.1. Viscosity

Polyol viscosity was measured using a Rotavisc lo-vi Complete, IKA, as described in ASTM D4878–15 [43], with a viscosity accuracy of 1%. The viscosimeter is provided with a temperature probe and a standard spindle SP-2 with a disc geometry with a diameter of 18.7 mm. The spindle was immersed into the polyol blend at a depth of 50 mm, and when room temperature was reached, the viscosity value was obtained. 

#### 2.3.2. Density

Polyurethane samples were cut into cylinders of 12 mm diameter and 12 mm height. Geometrical density was measured, as described in ASTM D1622/ D1622M-14 [44]. The values reported in this manuscript are the average value of three samples for each formulation.

#### 2.3.3. Cell Size

Foams were cut, and the growth plane (z) was examined by scanning electron microscopy with a FlexSEM 1000 Hitachi microscope. The energy of the electron beam was 10.0 kV. Prior to acquisition, the samples were coated with a gold monolayer to make them conductive. Cellular structures were analyzed by employing software based on Image J/FIJI [45] that allows one to determine the average cell size. The number of cells analyzed per sample was higher than 100. The normalized standard deviation (NSD) was also measured for each formulation as SD/cell size.

#### 2.3.4. Cell Density and Number of Nucleation Points

Considering the different relative densities of the samples when particles are added, it is necessary to calculate the cell nucleation density in order to make comparisons between the samples. 

Equation (1) [46] shows how cell density (*N_v_*) depends on porosity and cell size:

(1) $$N_{v}=\frac{6V_{f}}{\pi \Phi^{3}}$$

where *V_f_* is the porosity (1−*ρ_r_*) and *Φ* is the average cell size. The number of nucleation points per unit volume created in the solid material (Equation (2)) [46] (cell nucleation density) can be calculated by using the following equation:

(2) $$N_{0}=\frac{N_{v}}{\rho_{r}}$$

where *ρ_r_* is the relative density, the ratio between the density of the foam and the density of solid polymer (1160 kg m^−3^ for solid polyurethane). These two structural parameters were calculated for all the samples under study. 

## 3. Results and Discussion

### 3.1. Viscosity

**Figure 1:**
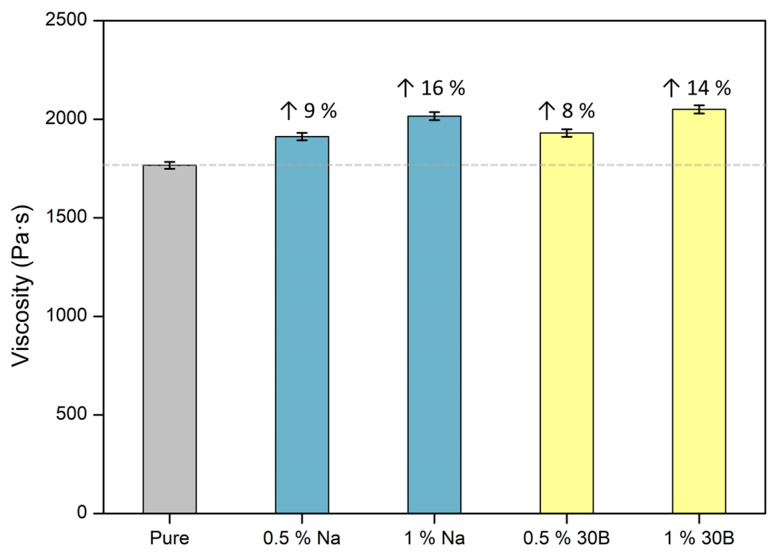
Viscosity values for the pure polyol blend and the polyol blend with the selected contents of both types of nanoclays.

Viscosity was measured for the reference polyol blend (containing catalysts, surfactant, and water) and for the polyol blend mixed with nanoclays after 5 min of stirring with the electric stirrer at 500 rpm. The effect of nanoclays on the final viscosity is shown in Figure 1. A similar effect was observed for both nanoparticle types. For the amount of fillers corresponding to the formulation with 0.5 wt%, the viscosity increased by ca. 8% with the addition of Clay Na^+^ and ca. 9% with Clay 30B with respect to the pure one. In the case of 1 wt% formulations, the viscosity rose by 14% and 16% for Clay Na^+^ and Clay 30B, respectively. Therefore, a significant increase in viscosity is detected even for a small number of particles.

### 3.2. Foam Density

Polyurethane densities and relative densities are listed in Table 5 for the formulations containing organomodified montmorillonite (Cloisite^®^ 30B) and natural sodium montmorillonite (Cloisite^®^ Na^+^).

Comparing the pure reference foams, geometrical densities were higher for the samples obtained with the magnetic stirrer, that is, M-A-Pure with 60.43 kg m^−3^ and M-V-Pure with 62.11 kg m^−3^, whereas with the electric stirrer, the density value was 44.18 kg m^−3^. This may be explained considering that stirring of the components with a magnetic stirrer is less effective than that obtained with an electric one. In the case of electric stirring, the addition of clays did not produce a significant modification of the final density (43.08 and 41.49 kg m^−3^ for 0.5 wt% and 1 wt% of Clay 30B, respectively, and 44.90 and 43.83 kg m^−3^ for 0.5 wt% and 1 wt% of Clay Na^+^, respectively). Nevertheless, regarding the magnetic-stirred formulations, the addition of 0.5 wt% or 1 wt% of Clay 30B led to a density increment for both atmospheric and vacuum fabrication conditions (M-A and M-V). For this type of clay, the density values were 67.57 and 72.22 kg m^−3^ for 0.5 wt% and 1 wt% under atmospheric conditions, respectively, and 61.26 and 73.27 kg m^−3^ for 0.5 wt% and 1 wt% when vacuum was applied, respectively.

The same effect was observed for electric-stirred foams when Clay Na^+^ was added (Table 5, bottom). Density continues to be constant despite the addition of natural clays under electric stirring conditions. However, when magnetic stirring is applied, the addition of these nanoclays promotes an increase in the final density, even for a lower content.

### 3.3. Cellular Structure

**Figure 2:**
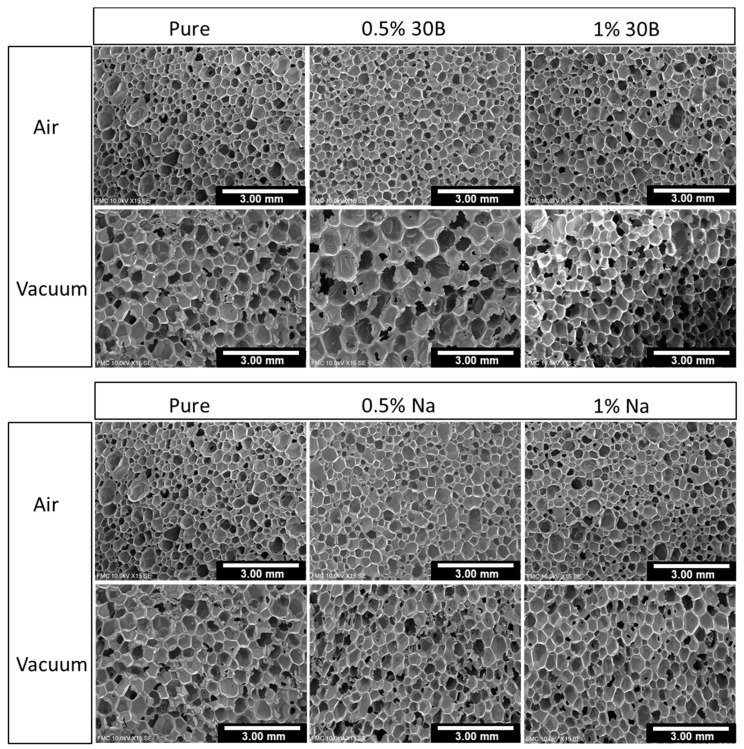
SEM micrographs of the polyurethane foams.

Micrographs of cellular structures are shown in Figure 2. Comparing the references (left columns), the cell size value of the foam fabricated under atmospheric conditions and with the electric stirrer (E-A-Pure) was 520 µm (Table 5). When using a magnetic stirrer (M-A-Pure), cells were slightly larger (570 µm), suggesting that more air is trapped in the blend by employing an electric stirrer. However, the cell size increased to 800 µm for the reference made under vacuum conditions. As expected, cells were notably larger when foams were fabricated under vacuum conditions, because less air was trapped during the isocyanate-polyol mixing. Therefore, a systematic comparison between the composites and the reference corresponding to its fabrication method was carried out in order to evaluate the nanoclay effect.

Figure 2 clearly shows how the cell size of E-A and M-A samples (those obtained under atmospheric conditions) notably decreased when particles were added to the reference. This effect was observed for both types of nanoclays (30B and Na^+^) and for both contents used in this work, decreasing from 520 µm to ca. 400 µm in the case of electric stirring and from 570 µm to ca. 500 µm for magnetic stirring (Table 5). As was discussed in the introduction, there are several studies where cell size reduction has been attributed to the addition of nanoclays [31,32,36]. However, considering the polyurethane foams manufactured under vacuum conditions (M-V), this cell size decrease is not as remarkable as that observed for foams produced using mixing at atmospheric pressure. In the case of vacuum mixing of materials containing nanoparticles, cell sizes are similar to those of the pure vacuum foam. This fact suggests that a nucleating effect exists when foams are produced by stirring under atmospheric conditions, but this does not occur under vacuum. Regarding the normalized standard deviation, it is higher for foams manufactured under atmospheric conditions and with an electric stirrer (E-A) due to the amount of air that is included into the mixture. When foams are mixed with a magnetic stirrer, this value is lower, indicating a higher homogeneity of the cellular structure for these foams.

**Figure 3:**
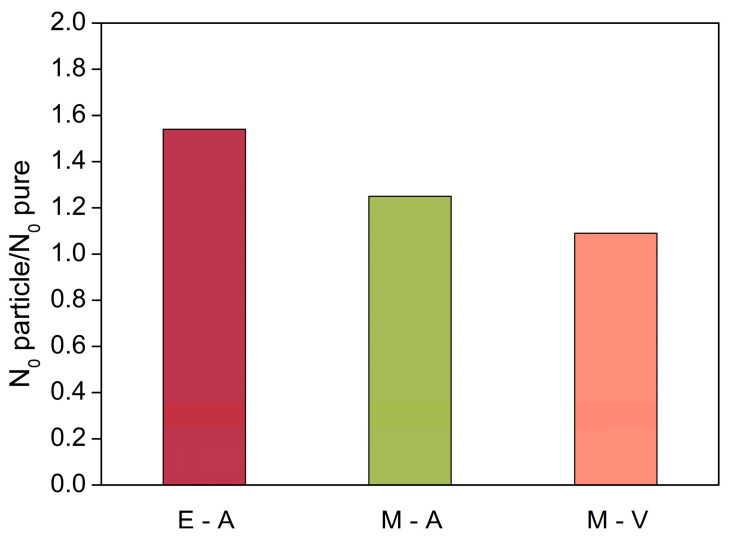
Ratio between the average nucleation density for samples with particles (30B and Na) and the corresponding pure foam for each fabrication method.

To analyze the nucleating effect, the cell size cannot be the only aspect taken into account, and the relative density must be also considered. As seen before, density presents a relationship with the stirring method. Thus, density has to be considered when comparing different nucleation levels. Due to this, we used the cell nucleation density, N_0_, as the key parameter for the comparison. This parameter showed an increase (Table 5) when the components were mixed under atmospheric conditions, which was more noticeable when the electric stirrer was used. For the M-V foams, the absence of air made this increment smoother. The ratio between the average cell nucleating density for the foams containing nanoclays (both Na^+^ and C30B) and the nucleation density for each corresponding reference was calculated in order to relate the nucleation effect for each fabrication method. These ratios are plotted in Figure 3, and they showed a clear increase in the nucleation centers for the PU foams stirred with the electric stirrer under atmospheric conditions (E-A) when nanoparticles were added. The ratio obtained for this manufacturing method was 1.54 (i.e., cell nucleation density increased in average by 1.54 times when the particles were added). Nevertheless, the foams that were obtained after mixing with a magnetic stirrer in the presence of air (M-A) showed a lower level of nucleation, owing to the lower amount of air trapped in the blend, giving a value of 1.25. Although the ratio was smaller than that obtained with electric stirring, a rise in the nucleation density took place with magnetic stirring since air was also present during the mixing steps. When vacuum conditions were reached (M-V), the nucleation density was similar to that of the reference foam, giving a ratio of 1.09. This value indicated that the addition of nanoparticles does not lead to a significant increase in the cell nucleation density when air inclusion is avoided during the stirring process.

## 4. Conclusions

Rigid PU foams with nanoclays as fillers were produced by using different manufacturing methods. The increase in the polyol viscosity was analyzed, showing a significant increase when nanoclays were included: natural nanoclays (Clay Na^+^) and organically modified nanoclays (Clay 30B) at different contents (0.5 and 1 wt%). The final density and cellular structures of the PU samples were studied in depth.

Given the possibility that two mechanisms can reduce the cell size in PU foams containing nanoclays, an experimental procedure was herein designed in order to elucidate which mechanism is more effective. The two possible mechanisms are as follows: 

Mechanism 1: Particles may reduce the surface tension, increasing the number of cells created when CO_2_ is produced.

Mechanism 2: The increase in the polyol blend viscosity containing the particles may promote more effective air trapping during mixing. The presence of a higher number of air bubbles, which act as nucleation sites in the reacting system, would lead to more effective nucleation.

The experimental approach consists of comparing the effect of the particles on the cell nucleation density when the mixing of components is carried out under atmospheric conditions or under vacuum. In this last case, air cannot be trapped and, therefore, the nucleating effect of the particles by a surface tension reduction is the only mechanism that could increase the number of cells in the foam.

The obtained results indicate that the more air is included during the mixing process, the higher is the nucleation effect. This is mainly due to the viscosity increase when particles are added, which leads to more effective air trapping. The obtained nucleation ratios for the procedures in which mixing was carried out under atmospheric conditions (E-A and M-A) were 1.54 and 1.25 for the electric and magnetic stirrers, respectively. However, under vacuum conditions (M-V), nanoclays do not present a nucleating effect and the nucleation density ratio was almost 1 (1.09). Therefore, it is clear that the nucleating effect, and the consequent reduction in cell size, when nanoparticles are added to rigid PU foams is mainly due to mechanism 2, i.e., the higher capacity of trapping air in the reactive blend when particles are added (through the increment in the blend viscosity).

## Figures and Tables

**Table 1:** Properties of the polyol used in this work.

Polyol Properties	
Polyol/isocyanate ratio	100/183
OH index (mg KOH g^−1^)	455
Polyol viscosity (25 °C) (mPa·s)	5250
Polyol molecular weight (g mol^−1^)	555
Polyol density (25 °C) (g cm^−3^)	1.08
Polyol water content (%)	0.1

**Table 2:** PU additives used in the production of PU foams expressed in parts per 100 parts of polyol weight (pphp).

Component	pphp
TEGOSTAB^®^ B 8522	1.0
PMDETA	0.3
TEGOAMIN^®^ DMCHA	1.0
Water	5.0

**Table 3:** Main characteristics of organomodified montmorillonite (Cloisite^®^ 30B) and natural sodium montmorillonite (Cloisite^®^ Na^+^).

Properties	Cloisite^®^ Na^+^	Cloisite^®^ 30B
Treatment	None	Methyl tallow-bis-2-hydroxyethyl quaternary ammonium
Density (g cm^−3^)	2.86	1.98
Loose bulk density (g cm^−3^)	0.20	0.23
Packed bulk density (g cm^−3^)	0.34	0.36
X-ray diffraction d-spacing (nm)	1.17	1.85

**Table 4:** Experimental details, compositions, and nomenclature of all the samples in the study.

Sample	Mixing Conditions	Stirrer Used	Clay 30B	Clay Na^+^
E-A-Pure	Air	Electric	-	-
M-A-Pure	Air	Magnetic	-	-
M-V-Pure	Vacuum	Magnetic	-	-
E-A-0.5% 30B	Air	Electric	0.5 wt%	-
M-A-0.5% 30B	Air	Magnetic	0.5 wt%	-
M-V-0.5% 30B	Vacuum	Magnetic	0.5 wt%	-
E-A-1% 30B	Air	Electric	1 wt%	-
M-A-1% 30B	Air	Magnetic	1 wt%	-
M-V-1% 30B	Vacuum	Magnetic	1 wt%	-
E-A-0.5% Na	Air	Electric	-	0.5 wt%
M-A-0.5% Na	Air	Magnetic	-	0.5 wt%
M-V-0.5% Na	Vacuum	Magnetic	-	0.5 wt%
E-A-1% Na	Air	Electric	-	1 wt%
M-A-1% Na	Air	Magnetic	-	1 wt%
M-V-1% Na	Vacuum	Magnetic	-	1 wt%

**Table 5:** Geometrical and relative densities for pure PU foams and Cloisite^®^ 30B or Cloisite^®^ Na^+^ filled foams fabricated under atmospheric and vacuum conditions, cell size values, and cell nucleation densities.

Sample	Density (kg m^−3^)	Relative Density	Cell Size (µm)	NSD	N_0_
E-A-Pure	44.18 ± 1.92	0.038	519.6	0.24	3.4 × 10^5^
M-A-Pure	60.43 ± 5.47	0.052	570.2	0.16	1.9 × 10^5^
M-V-Pure	62.11 ± 4.61	0.054	799.9	0.18	6.6 × 10^4^
E-A-0.5% 30B	43.08 ± 0.11	0.037	471.9	0.17	4.7 × 10^5^
M-A-0.5% 30B	67.57 ± 4.88	0.058	527.4	0.16	2.1 × 10^5^
M-V-0.5% 30B	61.26 ± 2.96	0.053	991.7	0.13	3.5 × 10^4^
E-A-1% 30B	41.49 ± 2.05	0.036	465.8	0.26	5.1 × 10^5^
M-A-1% 30B	72.22 ± 2.67	0.062	507.9	0.20	2.2 × 10^5^
M-V-1% 30B	73.27 ± 3.58	0.063	680.5	0.14	9.0 × 10^4^
E-A-0.5% Na	44.90 ± 0.76	0.039	496.6	0.25	3.9 × 10^5^
M-A-0.5% Na	71.51 ± 6.41	0.062	499.5	0.16	2.3 × 10^5^
M-V-0.5% Na	73.73 ± 1.05	0.064	688.2	0.16	8.6 × 10^4^
E-A-1% Na	43.83 ± 0.88	0.038	402.1	0.23	7.5 × 10^5^
M-A-1% Na	80.27 ± 3.61	0.069	479.6	0.18	2.3 × 10^5^
M-V-1% Na	77.62 ± 1.54	0.067	706.5	0.14	7.6 × 10^4^

## Data Availability

Not applicable.
